# The Presence of Residual Vascular and Adipose Tissue Inflammation on ^18^F-FDG PET in Patients with Chronic Coronary Artery Disease

**DOI:** 10.1007/s13139-022-00785-z

**Published:** 2022-12-26

**Authors:** Sini Toivonen, Miia Lehtinen, Peter Raivio, Juha Sinisalo, Antti Loimaala, Valtteri Uusitalo

**Affiliations:** 1 Department of Cardiac Surgery, Heart and Lung Center, Helsinki University Hospital and University of Helsinki, Helsinki, Finland; 2 Clinical Physiology and Nuclear Medicine, Helsinki University Hospital and University of Helsinki, Paciuksenkatu 3, 00290 Helsinki, Finland; 3 Department of Cardiology, Heart and Lung Center, Helsinki University Hospital and University of Helsinki, Helsinki, Finland; 4 Faculty of Medicine, Research Program in Systems Oncology, University of Helsinki, Helsinki, Finland

**Keywords:** Coronary artery disease, Inflammation, Positron emission tomography

## Abstract

**Purpose:**

We evaluated the residual vascular and adipose tissue inflammation in patients with chronic coronary artery disease (CAD) using positron emission tomography (PET).

**Methods:**

Our study population consisted of 98 patients with known CAD and 94 control subjects who had undergone ^18^F-fluorodeoxyglucose (^18^F-FDG) PET due to non-cardiac reasons. Aortic root and vena cava superior ^18^F-FDG uptake were measured to obtain the aortic root target-to-background ratio (TBR). In addition, adipose tissue PET measurements were done in pericoronary, epicardial, subcutaneous, and thoracic adipose tissue. Adipose tissue TBR was calculated using the left atrium as a reference region. Data are presented as mean ± standard deviation or as median (interquartile range).

**Results:**

The aortic root TBR was higher in CAD patients compared to control subjects, 1.68 (1.55–1.81) vs. 1.53 (1.43–1.64), *p* < 0.001. Subcutaneous adipose tissue uptake was elevated in CAD patients 0.30 (0.24–0.35) vs. 0.27 (0.23–0.31), *p* < 0.001. Metabolic activity of CAD patients and control subjects was comparable in the pericoronary (0.81 ± 0.18 vs. 0.80 ± 0.16, *p* = 0.59), epicardial (0.53 ± 0.21 vs. 0.51 ± 0.18, *p* = 0.38) and thoracic (0.31 ± 0.12 vs. 0.28 ± 0.12, *p* = 0.21) adipose tissue regions. Aortic root or adipose tissue ^18^F-FDG uptake was not associated with the common CAD risk factors, coronary calcium score, or aortic calcium score (*p* value > 0.05).

**Conclusion:**

Patients with a chronic CAD had a higher aortic root and subcutaneous adipose tissue ^18^F-FDG uptake compared to control patients, which suggests residual inflammatory risk.

## Introduction

Atherosclerosis progresses in a subset of patients with chronic coronary artery disease (CAD) despite guideline-recommended therapies resulting in myocardial infarctions, repeat revascularizations, and heart failure [1]. Some emerging strategies to reduce this residual risk include more intense anti-lipid therapy and dual anticoagulation [1–4]. Interestingly, residual inflammatory activity measured by high-sensitivity C-reactive protein (hs-CRP) is also common and associated with worse survival in patients with CAD [5, 6]. Moreover, recent clinical trials have shown potential for anti-inflammatory interventions in the treatment of CAD [7, 8].

Positron emission tomography (PET) using ^18^F-fluorodeoxyglucose (^18^F-FDG) is accurate in vivo modality for the measurement of vascular inflammation and metabolic activity [9, 10]. It allows direct measurement of vascular inflammation compared to laboratory biomarkers affected by the systemic inflammatory state. In previous studies, aortic ^18^F-FDG uptake has been related to an increased risk of cardiovascular disease in primary prevention settings [11, 12]. In addition, the carotid artery, adipose tissue, spleen, and bone marrow metabolic activity have been elevated in previous studies in patients with CAD [13, 14]. Thus, direct PET imaging of residual inflammation in chronic CAD might identify patients who benefit from additional secondary prevention using anti-inflammatory interventions.

In this study, we evaluate the presence of vascular inflammation and metabolic activity in different adipose tissue regions by PET imaging in chronic CAD patients. Moreover, we assess the related risk factors for high vascular or adipose tissue ^18^F-FDG uptake in patients with CAD.

## Methods

### Study Population

The study population consisted of 203 consecutive patients who had undergone ^18^F-FDG PET due to non-cardiac indications in Helsinki University Hospital between 2013 and 2015. Of these patients, 103 had been diagnosed with obstructive CAD. The other 100 patients included in this study were control subjects without known atherosclerotic disease for comparison of the average aortic PET signal in CAD patients to the reference population. Five patients with CAD were excluded from the study due to recent cardiac surgery (*n* = 2), recent myocardial infarction (*n* = 2), and known vasculitis (*n* = 1), which could affect the aortic metabolism. A total of 6 control patients were excluded due to peripheral artery disease (PAD, *n* = 2) or previous stroke (*n* = 4). Thus, the final study population consisted of 192 individuals shown in Table 1. Other exclusion criteria for the study were age < 50 years, non-diagnostic image quality, and metastatic cancer in control patients. Information on patients’ clinical characteristics was collected from the hospital’s records. Imaging indications for PET were classified as oncological, infectious or inflammatory, or miscellaneous. The intensity of statin treatment was defined according to previously accepted criteria by the American Heart Association [15]. The study was conducted according to the declaration of Helsinki. The study was approved by the local ethics committee (HUS/1226/2019), and the local institutional research board, and the need for written informed consent was waived.

**Table 1:** Patient characteristics at the time of PET imaging

Variable	No CAD *n* = 94	CAD *n* = 98	*p* value
Age (years)	64 ± 9	71 ± 6	< 0.001
Male	44 (46%)	77 (79%)	< 0.001
Coronary artery disease	0 (0%)	98 (100%)	
Indication for PET imaging			
Oncologic	92	83	0.003
Infection/autoimmune	1	14	
Other indication	1	1	
Active malignancy on PET	16 (17%)	43 (44%)	< 0.001
Previous revascularization	-	66 (67%)	
PCI	-	49 (50%)	
CABG	-	27 (28%)	
Previous myocardial infarct	-	60 (61%)	
Previous stroke	-	13 (13%)	
Peripheral artery disease	-	27 (28%)	
Heart failure	2 (2%)	37 (38%)	< 0.001
Renal failure	6 (6%)	26 (27%)	< 0.001
Hypertension	43 (45%)	87 (89%)	< 0.001
Hypercholesterolemia	36 (38%)	84 (86%)	< 0.001
Diabetes	17 (18%)	43 (44%)	< 0.001
Smoking	23 (24%)	31 (32%)	0.27
Body mass index (kg/m^2^)	25.6 (22.2 − 29.3)	25.8 (22.3 − 29.0)	0.84
Statin therapy	9 (10%)	77 (79%)	< 0.001
Blood pressure medication	39 (41%)	94 (96%)	< 0.001
Beta blocker	17 (18%)	80 (82%))	
ACE inhibitor / ATB	22 (23%)	57 (58%)	
Spironolactone	1 (1%)	9 (9%)	
Diuretic	13 (14%)	38 (39%)	
Calcium channel blocker	9 (10%)	28 (28%)	
Antiplatelet therapy	3 (3%)	75 (77%)	< 0.001
Anticoagulation	5 (5%)	14 (14%)	0.03

Data are presented as *n* (%), mean ± standard deviation, or median (interquartile range). *ATB* angiotensin II receptor blocker; angiotensin-converting enzyme, *CABG* coronary artery bypass surgery, ^*18*^*F-FDG* fluorodeoxyglucose, *PCI* percutaneous coronary intervention

### Positron Emission Tomography

Patients were advised to fast for at least 8 h before the PET imaging. Blood glucose level was measured before the imaging, and when < 10 mmol/l, the study was performed. After intravenous ^18^F-FDG injection, patients rested for 60 min in a quiet and semi-darkened room. A standard clinical PET camera was used for the imaging (Gemini PET-CT scanner, Philips Inc, USA). PET images were retrospectively analyzed for aortic root ^18^F-FDG activity as previously described [12]. A total of 6 to 8 regions of interest were drawn to ascending aorta and 10 to 12 to the vena cava superior, respectively. The average maximal standardized uptake value (SUVmax) of each region of interest slice in ascending aorta was used. The mean standardized uptake value of vena cava superior was used to correct for the blood radioactivity. The aortic root’s target-to-background ratio (TBR) was calculated by dividing aortic SUVmax with the mean SUV value in the superior vena cava. If the superior vena cava was judged as non-reliable for blood pool analysis, the inferior vena cava or right atrial blood pool was selected for comparison. Pericoronary, epicardial, subcutaneous, and thoracic mediastinal adipose tissue ^18^F-FDG uptake was measured as previously described [16]. In brief, pericoronary adipose tissue measurement was an average of left ascending, left circumflex, and right coronary artery regions. Subcutaneous fat measurements were an average of the anterior and posterior subcutaneous regions at the level of the sternum. Thoracic adipose tissue measurement was done retrosternally. The mean SUV value of the left atrium was used as a reference to calculate adipose tissue TBR. PET analyses were performed using Hermes software (HERMES Medical Solutions, Stockholm, Sweden).

### Calcium Score

Agatston calcium scores of the ascending aorta and coronary arteries were measured from low-dose non-electrocardiograph gated computed tomography (CT) images obtained with PET/CT imaging. Coronary calcium score (CCS) was categorized as low (0–100), moderate (100–400), and extensive (> 400). The OsiriX software was used for the calcium score analysis (OsiriX DICOM Viewer, Pixmeo SARL, Geneve, Switzerland).

### Statistical Analysis

Parametric continuous variables were described as mean ± standard deviation and non-parametric as median (interquartile range). Categorical variables were presented as numbers and percentages. All continuous variables were treated as continuous in statistical analyzes unless otherwise specified. Parametric continuous variables were compared using Student’s *t*-test and one-way analysis of variance with the Bonferroni correction for multiple comparisons. Non-parametric variables were studied using the Mann–Whitney U test and the Kruskal–Wallis one-way analysis of variance. Categorical variables were analyzed using the chi-squared test of independence. A comparison of clinical and laboratory variables with aortic PET signal was made using multiple linear regression. Two-tailed *p* values of < 0.05 were considered statistically significant. Statistical analyses were performed using the SPSS statistics 27 (SPSS Inc, Chicago, IL).

## Results

### Baseline Patient Characteristics

The final study population comprised 192 individuals, of whom 98 had chronic CAD. Patient characteristics are summarized in Table 1. Diagnosis of CAD was based on invasive angiography (*n* = 78), history of previous myocardial infarction (*n* = 1), single-photon emission computed tomography (*n* = 2), or exercise electrocardiogram (*n* = 2). In 15 individuals, the method for CAD diagnosis was not available in the hospital’s electronic health records. The median time between the diagnosis of CAD and PET scan was 7 years (1–18 years). Myocardial infarctions in CAD patients occurred a median of 7 years (1–16) years before PET imaging.

**Fig. 1:**
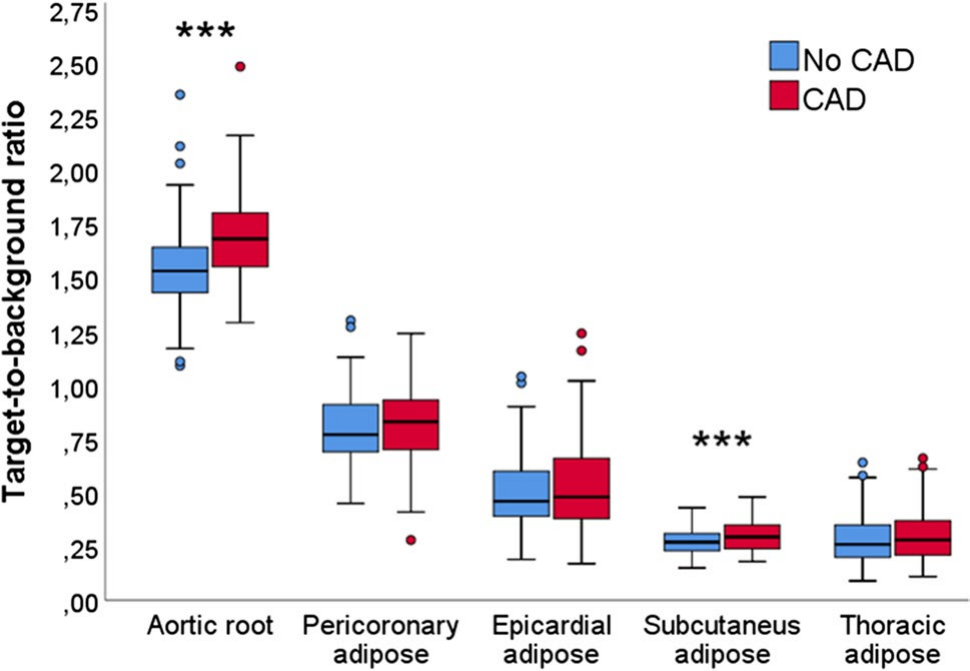
Comparison of vascular and adipose tissue.^18^F-fluorodeoxyglucose activity on positron emission tomography in patients with and without chronic coronary artery disease (CAD). ***p < 0.001

As shown in Fig. 1, the aortic root and the subcutaneous adipose tissue ^18^F-FDG uptake was higher in patients with CAD than in control subjects. In contrast, the metabolic activity in pericoronary, epicardial and thoracic adipose tissue regions was similar between both groups. There was no difference in aortic TBR in patients with oncologic (*n* = 175), infectious or inflammatory (*n* = 15), or other miscellaneous (*n* = 2) indications for PET imaging 1.59 (1.48–1.71) vs. 1.61 (1.56–1.87) vs. 1.78, *p* = 0.24), respectively. Infectious or inflammatory indication for PET was not related to higher aortic root activity compared to other imaging indications (1.64 (1.55–1.87) vs. 1.69 (1.54–1.79), *p* = 0.94). Similarly, the adipose tissue metabolic activity in pericoronary, epicardial, subcutaneous, and thoracic adipose tissue regions was comparable in patients with different imaging indications for PET imaging (*p* value > 0.05).

### Predictors of Aortic ^18^F-FDG Uptake in CAD

**Fig. 2:**
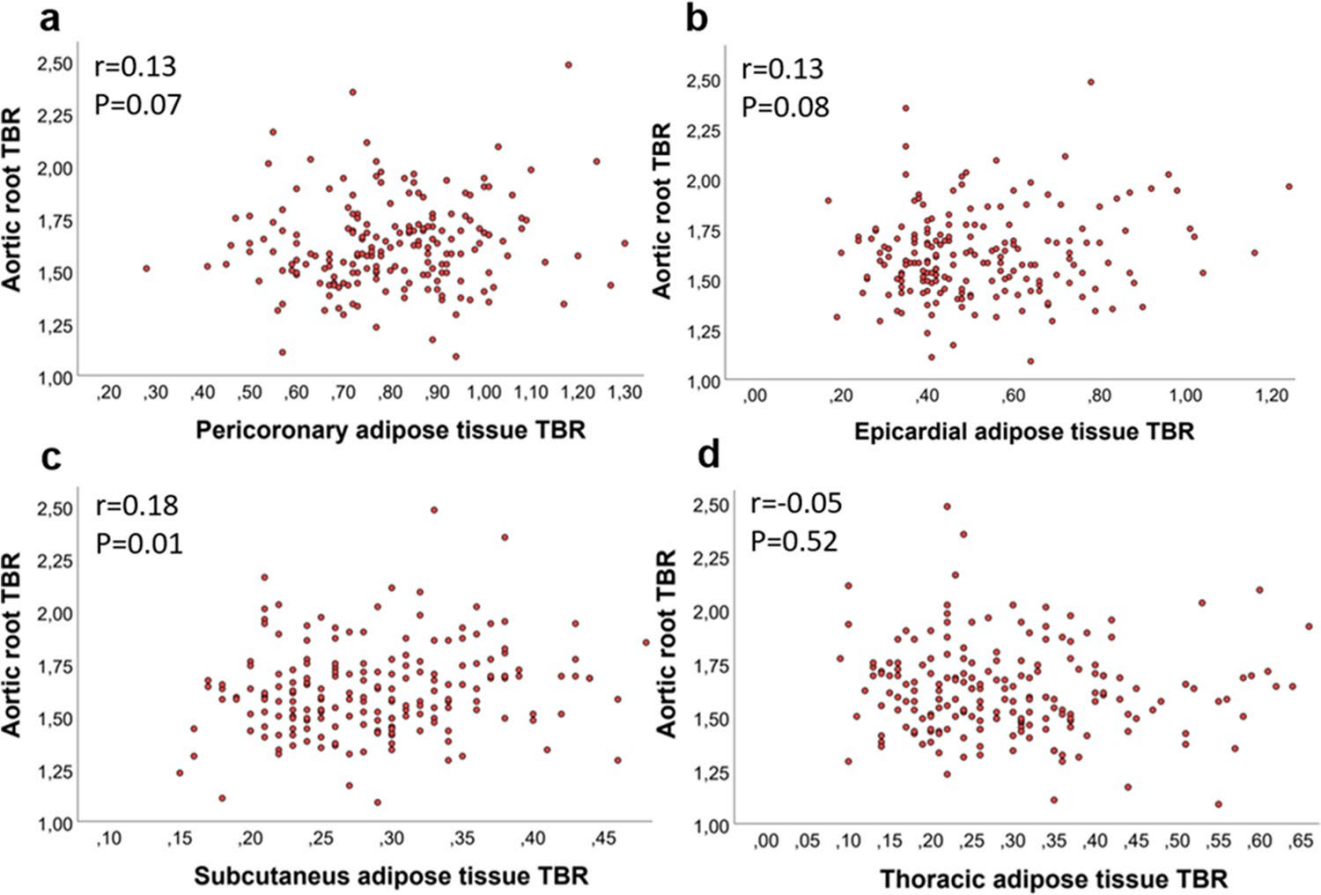
The relationship between the intensity of aortic root and adipose tissue ^18^F-fluorodeoxyglucose uptake in pericoronary (**a**), epicardial (**b**), subcutaneous (**c**), and thoracic regions (**d**). TBR target-to-background ratio

Univariate predictors of the aortic TBR ratio are shown in Table 2. Of note, older age and a history of peripheral artery disease were associated with a lower aortic ^18^F-FDG activity. Other patient characteristics, including CAD risk factors, previous myocardial infarction, heart failure, or stroke, were not significantly related to aortic ^18^F-FDG uptake. Similarly, blood leukocyte count or C-reactive protein levels were not associated with aortic PET signal (*p* value > 0.05). There was no relationship between the intensity of statin treatment or prior coronary revascularization with aortic root ^18^F-FDG activity, as shown in Fig. 2. Only four CAD patients had no prescribed blood pressure medications at the time of PET imaging, and they had elevated aortic TBR compared to other patients (1.90 (1.75–2.00) vs. 1.68 (1.54 vs. 1.77), *p* = 0.03). Use of angiotensin-converting enzyme inhibitors, angiotensin II receptor blockers, or beta blockers did not affect aortic ^18^F-FDG uptake (1.70 (1.57–1.83) vs. 1.68 (1.53–1.81), *p* = 0.56 and 1.71 (1.57–1.88) vs. 1.67 (1.55–1.80), *p* = 0.42). None other blood pressure medications, antiplatelet therapy, or anticoagulation were related to aortic inflammation (data not shown).

**Table 2:** Univariate predictors of the degree of aortic root inflammation measured as target-to-background ratio on ^18^F-FDG PET in patients with chronic CAD

Variable	Coefficient	95% CI	*p* value
Age (years)	− 0.01	− 0.01; − 0.002	0.03
Female	− 0.04	− 0.14; 0.06	0.40
Diabetes	− 0.07	− 0.15; 0.02	0.11
BMI (kg/m2)	− 0.01	− 0.01; 0.002	0.18
Hypertension	− 0.09	− 0.21; 0.04	0.18
Total cholesterol (mmol/l)	− 0.01	− 0.06; 0.04	0.64
LDL (mmol/l)	− 0.02	− 0.07; 0.03	0.47
HDL (mmol/l)	0.09	− 0.02; 0.20	0.11
Triglycerides (mmol/l)	− 0.03	− 0.09; 0.02	0.26
GFR (ml/min)	0.001	− 0.0003; 0.002	0.17
History of autoimmune disease	0.05	− 0.05; 0.14	0.33
Smoking	0.04	− 0.04; 0.14	0.27
COPD	− 0.06	− 0.14; 0.04	0.24
Peripheral artery disease	− 0.10	− 0.18; − 0.01	0.03
Previous PCI	− 0.02	− 0.05; 0.01	0.31
Previous CABG	− 0.02	− 0.02; 0.05	0.33
Previous myocardial infarct	0.04	− 0.05; 0.12	0.37
Previous stroke	− 0.04	− 0.17; 0.08	0.52
Heart failure	− 0.0003	− 0.08; 0.08	1.00
No statin medication	0.02	− 0.08; 0.12	0.69

*BMI* body mass index, *COPD* chronic pulmonary obstructive disease, ^*18*^*F-FDG PET* fluorodeoxyglucose positron emission tomography, *CABG* coronary artery bypass graft surgery, *GFR* glomerular filtration rate, *HDL* high density lipoprotein, *LDL* low-density lipoprotein, *PCI* percutaneous coronary intervention, *TBR* target-to-background ratio

### Vascular Inflammation and the Extent of Atherosclerosis in CAD

The degree of aortic inflammation showed no difference between CAD patients with low, moderate, or high CCS (1.71 (1.59–1.76) vs. 1.64 (1.49–1.69) vs. 1.70 (1.55–1.87), *p* = 0.17). Of note, in the control group, seven individuals had moderate (CCS 100–400), and 3 had extensive coronary atherosclerosis (CCS > 400). The presence of greater than moderate coronary calcification in control subjects was not related to higher aortic ^18^F-FDG uptake (1.53 (1.43–1.64) vs. 1.52 (1.35–1.68), *p* = 0.78). The aortic ^18^F-FDG activity was not related to the degree of CCS or aortic calcification in patients without diagnosed CAD (95% CI − 2.51; 1.58, *p* = 0.65 and 95% CI − 3.15; 1.09, *p* = 0.09).

### Adipose Tissue ^18^F-FDG Uptake and its Related Risk Factors

**Fig. 3:**
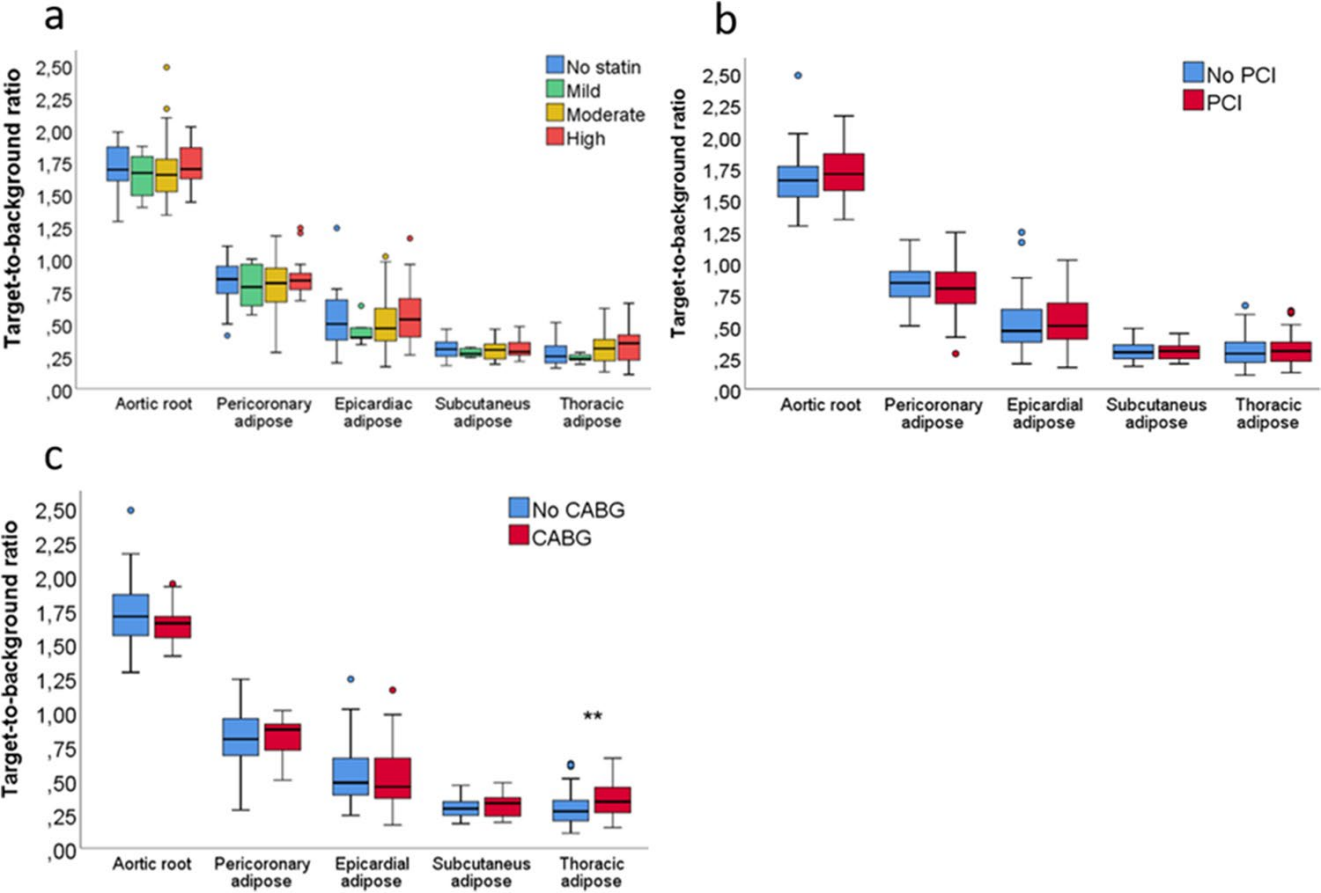
Impact of statin treatment and previous revascularization on the vascular and adipose tissue ^18^F-fluorodeoxyglucose (^18^F-FDG) uptake in patients with chronic coronary artery disease. **a** Vascular and adipose tissue metabolic activity in patients with different intensities of statin treatment. **b** Vascular and adipose tissue.^18^F-FDG uptake in patients with previous percutaneous coronary intervention (PCI). **c** The effect of previous coronary artery bypass graft (CABG) surgery on vascular and adipose tissue metabolic activity. **p < 0.01

As shown in Fig. 1, the subcutaneous ^18^F-FDG uptake was elevated in CAD patients compared to control subjects. Metabolic activity in the subcutaneous region also correlated significantly with the aortic root activity, as shown in Fig. 2. The presence of hypercholesterolemia, hypertension, diabetes, obesity (body mass index > 30 m^2^/kg), smoking, or coronary calcium score was not related to higher adipose tissue PET uptake in any of the measured areas in patients with CAD in the linear regression analysis (*p* value always > 0.05). As depicted in Fig. 3, there was no difference in adipose tissue ^18^F-FDG uptake in patients receiving different doses of statin therapy or in CAD patients with or without previous revascularization by PCI. Patients with prior CABG had higher thoracic adipose tissue TBR. The history of myocardial infarction or heart failure was unrelated to adipose tissue metabolic activity in any measured regions (*p* value always > 0.05). Table 3. characterizes patients with both elevated vascular and adipose tissue PET uptake.

**Table 3:** The clinical differences in coronary artery disease patients with both aortic and subcutaneous target-to-background ratios above the median ^18^F-FDG uptake (PET +) and other patients (PET −)

Variable	PET − *n* = 65	PET + *n* = 33	*p* value
Age (years)	72 ± 6	69 ± 7	0.52
Male	51 (78%)	26 (79)	0.97
Previous revascularization	24 (37%)	8 (24%)	0.21
PCI	30 (46%)	19 (58%)	0.29
CABG	17 (26%)	10 (30%)	0.66
Previous myocardial infarct	38 (58%)	22 (33%)	0.43
Previous stroke	8 (12%)	4 (12%)	0.98
Peripheral artery disease	20 (31%)	8 (24%)	0.50
Heart failure	26 (40%)	11 (33%)	0.52
Renal failure	17 (26%)	9 (27%)	0.91
Hypertension	58 (89%)	29 (88%)	0.84
Hypercholesterolemia	54 (83%)	30 (91%)	0.30
Body mass index (kg/m^2^)	25.8 (22.8 − 29.2)	25.6 (21.3 − 28.7)	0.58
Diabetes	32 (49%)	11 (33%)	0.13
Smoking	20 (31%)	11 (33%)	0.80
COPD	22 (34%)	6 (22%)	0.11
Autoimmune disease	18 (28%)	6 (18%)	0.30
Coronary calcium score	558 (167 − 1793)	656 (166 − 1262)	0.73
Aortic calcium score	155 (11 − 679)	45 (13 − 552)	0.75
Statin therapy	51 (80%)	26 (79%)	0.92

Data are presented as *n* (%), mean ± standard deviation, or median (interquartile range); *CABG* coronary artery bypass surgery, *COPD* chronic obstructive pulmonary disease, *PCI* percutaneous coronary intervention

## Discussion

This study evaluated the aortic root and adipose tissue metabolic activity on ^18^F-FDG-PET in patients with chronic CAD. Detection of residual vascular or adipose tissue inflammation in CAD patients could guide more intensive secondary prevention and novel anti-inflammatory interventions. Our main finding is that patients with CAD had greater aortic root and subcutaneous adipose tissue ^18^F-FDG uptake than control subjects, suggestive of potentially treatable residual inflammatory risk.

### Vascular Inflammation in CAD

Previous research has mainly focused on aortic inflammation on ^18^F-FDG PET as a risk factor for primary prevention in patients without the diagnosed cardiovascular disease [11, 12]. In contrast, we studied patients with chronic CAD who are more clinically feasible targets for novel, expensive, anti-inflammatory treatments in addition to a normal treatment regimen. Surprisingly, vascular ^18^F-FDG uptake in CAD patients was not associated with cardiovascular risk factors or laboratory biomarkers in our study. Moreover, previous myocardial infarction or revascularization was unrelated to aortic root metabolism. Previous studies have demonstrated the importance of inflammatory risk measured by either hs-CRP or PET as a risk factor for future myocardial infarction and mortality [6, 11, 12]. In addition, metabolic activity on PET is frequent in high-risk coronary plaques and in carotid arteries of patients with previous myocardial infarction [13, 14, 17, 18]. Interestingly, anti-inflammatory therapeutic response to preventive medications can also be monitored by PET [19]. This discrepancy with our results is understandable as the relationship between inflammation, short-term progression of atherosclerotic plaques, and clinical adverse events is not straightforward [20, 21]. Firstly, our CAD population is heterogeneous and represents patients at different stages of the atherosclerotic process, while vascular inflammation seems to be most pronounced with early disease [22]. Indeed, age and the presence of PAD were inversely correlated with vascular uptake in our study. Patients with both CAD and PAD might also receive more intensive secondary prevention and clinical monitoring due to their high-risk status. Older individuals might also have selection bias due to survival in our study. Furthermore, myocardial infarction at advanced CAD can be caused by intimal erosion rather than fibrous cap rupture of vulnerable plaque with a high metabolism [23]. Secondly, clinical risk calculators in individual patients are known to be poor predictors of the extent of atherosclerosis and of future cardiac events, of which a significant proportion occurs in individuals deemed as having low risk [24, 25]. Thus, PET's direct measurement of vascular inflammation might better identify patients vulnerable to atherosclerosis progression and who benefit from additional interventions.

### Adipose Tissue Metabolism in CAD

We observed elevated subcutaneous adipose tissue activity in CAD patients compared to control subjects. Similarly to our results, Takx et al. have demonstrated a relationship between vascular inflammation and brown fat metabolism in the primary prevention population. In their study, reduced brown fat ^18^F-FDG uptake was related to a higher risk for cardiovascular events [26]. Our results also agree with a previous study demonstrating elevated visceral fat activity in patients with chronic CAD and acute myocardial infarction [14]. In addition to vascular metabolism, adipose tissue PET activity seems to relate to myocardial glucose utilization, which could influence myocardial function [27]. Subcutaneous adipose tissue ^18^F-FDG uptake is a simple and fast measure and is not significantly affected by cardiac or respiratory movement, partial volume effect, or adjacent ^18^F-FDG hot spots. Moreover, novel diabetes treatments have demonstrated a survival benefit in CAD patients, which could suggest adipose tissue metabolism as a clinically feasible target for guiding secondary prevention [28]. A combination of both vascular and adipose tissue PET activity was explored in our study, but we did not see any additional value in the combined analysis of both. PET uptake in other adipose tissue regions, including pericoronary, epicardial, and intrathoracic adipose tissue, was comparable in individuals with and without CAD. The subcutaneous adipose region might be an easier and more feasible imaging target for residual inflammatory risk compared to the aortic root or other vascular regions. Furthermore, the adipose tissue region would still be a viable analysis target in patients with aortic prosthesis or other factors confounding the vascular PET analysis. Still, its reference values should be established in more extensive studies in the future. Notably, retrosternal thoracic adipose tissue uptake was elevated in patients with previous CABG, which might be related to the surgical operation or secondary to sternal wire attenuation artifact.

### Prospects of Clinical ^18^FDG-PET Imaging in CAD

The prognostic value of anti-inflammatory intervention using canakinumab after myocardial infarction has been demonstrated in a recent study by Ridker et al. [7]. History of myocardial infarct was not associated with greater inflammatory burden in our heterogenous CAD population, suggesting that the optimal time-point for vascular PET imaging might be early after infarction to screen for high-risk individuals for expensive monoclonal antibody intervention. Nonetheless, the study by Nidorf et al. has shown that at least a subpopulation of chronic CAD patients also benefits from anti-inflammatory treatment [8]. Thus, PET imaging targeted secondary prevention seems possible in chronic CAD but waits for future clinical validation. Similarly, to our study, patients with ^18^FDG-PET imaged due to non-cardiac indication might be a feasible setting for aortic or adipose tissue activity measurements for early primary prevention interventions or escalation of secondary prevention in patients with established cardiovascular disease.

### Limitations

Our study population was retrospectively collected and consisted of a heterogenic population of CAD patients. The definition of CAD in our study was based on patient records by a cardiologist. However, the majority of the diagnoses were done by invasive angiography (80%). CAD patients had more inflammatory indications for PET imaging than others, but it was not associated with greater aortic or adipose tissue TBR. Individual adherence to prescribed preventive medications might vary, which might influence our results. PET imaging was done 1 h after radiotracer injection, which might not be the optimal time-point for vascular PET. Oncological treatments might have an impact on vascular PET signal. Therefore, our study included a similar control group of patients without CAD. We did not evaluate the spleen or bone marrow metabolism in our study as they are impacted by the oncological disease and its treatments. Since PET scans were not ECG-gated and were obtained during free breathing, we could not directly measure coronary ^18^F-FDG uptake. However, aortic root or adipose tissue metabolism might be better suited to reflect patients’ vascular inflammation at the systemic level. Aortic and coronary calcium score was also obtained from images without ECG-gating. Further studies are needed to establish the normal range of vascular and adipose tissue ^18^FDG-PET measures in primary and secondary prevention settings to enable clinical interventions.

## Conclusions

Patients with chronic CAD had a higher aortic root and subcutaneous adipose tissue ^18^F-FDG uptake compared to control patients, which suggests residual inflammatory risk and potential for increased secondary prevention. Metabolic activity in pericoronary, epicardial, or thoracic adipose tissue regions was comparable in patients with and without CAD.

## Data Availability

Contact the corresponding author for data requests.
